# Saunders’s Question Compends

**Published:** 1903-02

**Authors:** 


SAUNDERS'S QUESTION COMPENDS. Essentials of Diseases of the Ear. By E. B. GLEASON, S. B., M. D., Clinical Professor of Otology, Medico-Chirurgical College, Philadelphia; Surgeon in charge of the Nose, Throat and Ear Department of the Northern Dispensary, Philadelphia. Third edition, thoroughly revised 16mo volume of 214 pages, with 114 illustrations. Philadelphia and London: W. B. Saunders & Co. 1902. [Cloth, $1.00 net.]

These little books have always been admirable and this one is no exception. Their standard of excellence was established years ago and is so well known that further comment upon it is unnecessary; the only difference between the new and the old being the additions required to bring the subjects down to date. In that respect Dr. Gleason has left nothing undone. L. B.

